# Antimutagenic Compounds of White Shrimp (*Litopenaeus vannamei*): Isolation and Structural Elucidation

**DOI:** 10.1155/2016/8148215

**Published:** 2016-02-24

**Authors:** Carmen-María López-Saiz, Javier Hernández, Francisco-Javier Cinco-Moroyoqui, Carlos Velázquez, Víctor-Manuel Ocaño-Higuera, Maribel Plascencia-Jatomea, Maribel Robles-Sánchez, Lorena Machi-Lara, Armando Burgos-Hernández

**Affiliations:** ^1^Departamento de Investigación y Posgrado en Alimentos, Universidad de Sonora, Apartado Postal 1658, 83000 Hermosillo, SON, Mexico; ^2^Programa de Ingeniería Ambiental, Universidad Estatal de Sonora, 83000 Hermosillo, SON, Mexico; ^3^Unidad de Servicios de Apoyo en Resolución Analítica, Universidad Veracruzana, 91240 Xico, VER, Mexico; ^4^Departamento de Ciencias Químico-Biológicas, Universidad de Sonora, 83000 Hermosillo, SON, Mexico; ^5^Departamento de Investigación en Polímeros y Materiales, Universidad de Sonora, 83000 Hermosillo, SON, Mexico

## Abstract

According to the World Health Organization, cancer is the main cause of mortality worldwide; thus, the search of chemopreventive compounds to prevent the disease has become a priority. White shrimp (*Litopenaeus vannamei*) has been reported as a source of compounds with chemopreventive activities. In this study, shrimp lipids were extracted and then fractionated in order to isolate those compounds responsible for the antimutagenic activity. The antimutagenic activity was assessed by the inhibition of the mutagenic effect of aflatoxin B_1_ on TA98 and TA100* Salmonella* tester strains using the Ames test. Methanolic fraction was responsible for the highest antimutagenic activity (95.6 and 95.9% for TA98 and TA100, resp.) and was further separated into fifteen different subfractions (M1–M15). Fraction M8 exerted the highest inhibition of AFB_1_ mutation (96.5 and 101.6% for TA98 and TA100, resp.) and, after further fractionation, four subfractions M8a, M8b, M8c, and M8d were obtained. Data from ^1^H and ^13^C NMR, and mass spectrometry analysis of fraction M8a (the one with the highest antimutagenic activity), suggest that the compound responsible for its antimutagenicity is an apocarotenoid.

## 1. Introduction

In economically developed countries, cancer, a disease considered preventable [1], has been reported as the leading cause of death and second in developing countries [2]. Cancer prevention can be mainly achieved through life style changes which may include the chemopreventive and chemoprotective compounds in the diet. Chemopreventive agents are able to reverse, suppress, or prevent the cancer development [3]. Naturally occurring bioactive extracts or compounds have been reported to be beneficial for human health by inhibiting carcinogenic processes [4, 5]. One of these biological activities is antimutagenicity, which is given by compounds that have the ability to offer protection against induced DNA mutation [6–8]. This bioactivity could be given by different mechanisms of action, such as prevention of conversion of a promutagen into mutagenic compounds (bioactivation inhibition), reaction with the mutagen (mutagen blockade) preventing the interaction with DNA, and the stimulation of damaged DNA repairing systems [9]. In the search for these kinds of compounds, more than fifteen thousand natural compounds and extracts have been isolated from different seafood [10] and tested for different biological activities [11], including shrimp.

Shrimp muscle has been reported as a rich source of high quality proteins and also low in fat content [12, 13] and even though this lipidic fraction only accounts for a small percentage, there is convincing evidence that it may exhibit different biological activities. Previous reports have determined the presence of antioxidant [14–16] and anti-inflammatory [16] compounds in different byproducts (head and exoskeleton) of some shrimp species and also antimutagenic activity in their muscle [17, 18]; nevertheless, the chemical nature of these compounds has not been determined yet.

The lipidic fraction of shrimp muscle contains different compounds including neutral lipids, phospholipids, glycolipids, and carotenoids [19]. This fraction accounts for 1-2% of muscle weight (dry weight) [19]. In the search for antimutagenic activity, several individual carotenoids including* meso*-zeaxanthin, *β*-carotene, zeaxanthin, *α*-carotene, and astaxanthin and its esters have been, individually or in combination, tested by the Ames test [20] finding them capable of inhibiting known carcinogenic compounds (such as ethidium bromide, sodium azide, and hydroxyl amine). The aim of this study was to isolate and identify the antimutagenic compounds responsible for shrimp muscle high antimutagenic activity.

## 2. Materials and Methods

### 2.1. Testing Species

White shrimp (*Litopenaeus vannamei*) was purchased from the local market at Hermosillo, Sonora, Mexico, and transported in ice to the laboratory. Shrimp muscle was obtained, packed in self-sealing polyethylene bags, and stored at –20°C until their use. Shrimp muscle lipid fraction was extracted according to López-Saiz et al. [21].

### 2.2. Lipid Composition Analysis by RP-HPLC

**Figure 1:**
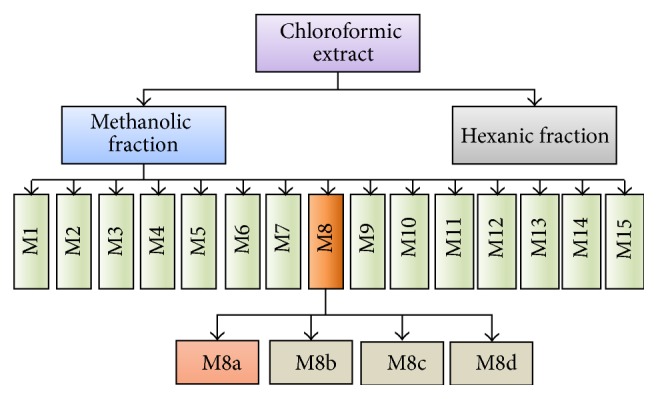
Schematic for the isolation of antimutagenic compounds from shrimp.

Fractionation (Figure 1) of white shrimp muscle lipidic extract was carried out according to López-Saiz et al. [21]. The antimutagenicity activity was individually analyzed in every chromatographic fraction collected.

### 2.3. Open Column Chromatography

The subfraction with the highest antimutagenic activity was further fractionated using open column chromatography on silica gel (2.5 cm × 60 cm), using 230–400-mesh silica gel (Sigma-Aldrich, St. Louis, MO, USA). Subfraction M8 was poured onto the column and eluted using 500 mL of a series of mobile phases as follows: (A) hexane : ethyl acetate (8 : 2), (B) hexane : ethyl acetate (7 : 3), (C) hexane : ethyl acetate (2 : 3), (D) hexane : ethyl acetate (1 : 1), (E) ethyl acetate : hexane (4 : 1), (F) acetone, and finally (G) methanol. Silica gel-coated TLC testing plates, revealed with an iodide solution and observed under UV light, were used to monitor the eluents. Fractions providing similar signals were combined and used for further analyses.

### 2.4. Bacterial Cultures

Overnight* Salmonella typhimurium* TA98 and TA100 tester strain cultures were stored at −80°C. Tester strains genetic characteristics were periodically confirmed according to Maron and Ames [22].

### 2.5. Antimutagenicity Test

The* Salmonella*/microsomal mutagenicity test [22] was used to assess the antimutagenicity of crude extracts and chromatographic fractions, according to the protocol reported by Wilson-Sanchez et al. [18], using acetone to reconstitute fractions to concentrations of 40 or 50 mg/mL. All assays were carried out in triplicate.

Antimutagenic activity was reported as the percentage of AFB_1_ inhibition according to the following equation:

(1) $$\begin{array}{c} \%\;\;Antimutagenicity=\frac{TR}{{AFB}_{1}R}\times 100, \end{array}$$

where TR is number of treatment-induced revertants/plate and AFB_1_R is number of aflatoxin B_1_-induced revertants/plate (positive control).

### 2.6. ^1^H and ^13^C NMR Analysis

Analyses were carried out using Agilent Technologies 400/54 Premium Shielded (400 MHz) spectrometers. A 500 *µ*L aliquot of CDCl_3_ (Sigma-Aldrich, Saint Louis, Missouri, USA) was used to dissolve each fraction and tetramethylsilane (TMS) was also included as an internal standard. This mixture was placed into 5 mm diameter ultraprecision NMR sample tubes. Chemical shifts were registered as ppm units, employing TMS proton signals as internal standard.

### 2.7. Statistical Analysis

Data treatment was carried out using one-way analysis of variance (ANOVA) using Tukey-Kramer multiple comparison of means (Number Cruncher Statistical Software (NCSS), Kaysville, UT, USA) with a significance level of *P* ≤ 0.05.

## 3. Results and Discussion

### 3.1. Lipidic Extraction and Partition

Chloroform extraction from shrimp muscle yielded 1.860 ± 0.004% (dry basis), a value that falls within the lipid content (1-2% of its dry weight) that has been previously reported [19].

Antimutagenic activity was assessed with the standard Ames test, using aflatoxin B_1_ (AFB_1_) as control mutagen. Shrimp muscle chloroform-extract inhibited AFB_1_ mutagenic potential in 94.6 ± 1.1 and 95.36 ± 2.41% in both* Salmonella typhimurium* TA98 and TA100 tester strains, respectively (Table 1). These results suggested the presence of compounds that are highly capable of inhibiting AFB_1_ [23]. Antimutagenic activity had previously been reported for shrimp flesh, using sodium azide and potassium permanganate [17] and also AFB_1_ [18] as control mutagens.

#### 3.1.1. Antimutagenic Activity of Partitioned Fractions

The lowest antimutagenic activity against AFB_1_ was exerted by the hexanic fraction while the methanolic fraction showed the highest (95.6 ± 0.6 and 95.9 ± 1.9% for TA98 and TA100 tester strains, resp.), which was comparable to that obtained for the chloroform-extract (Table 1). Based on the above, the methanolic fraction was subjected to further fractionation.

### 3.2. Lipid Composition Analysis by RP-HPLC

**Figure 2:**
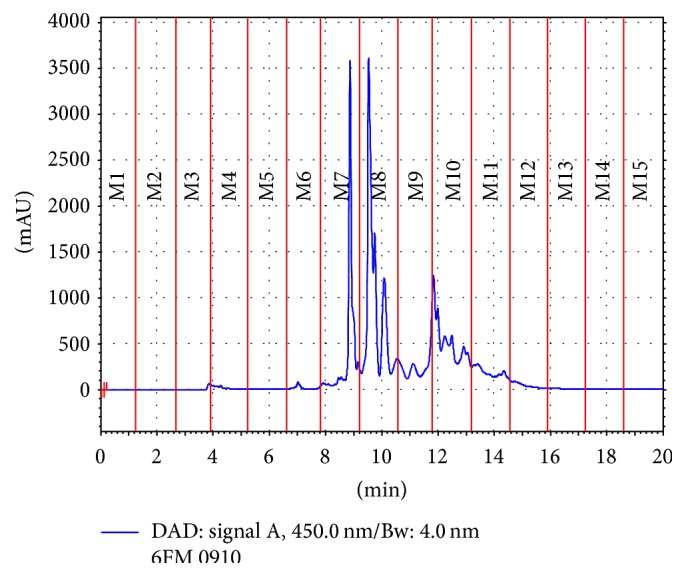
RP-HPLC analysis and fractionation of methanolic fraction (absorbance at 450 nm).

The methanolic fraction was separated into 15 different subfractions according to their retention times. The highest absorbance registered for the methanolic fraction was at 450 nm (Figure 2), signals that usually are attributed to carotenoid compounds found in muscle of shrimp [24]; these compounds include astaxanthin [24] and, at lower amounts, astaxanthin esters [25, 26]. *α*-Carotene, *β*-cryptoxanthin, *β*-carotene [27], lutein, canthaxanthin, and zeaxanthin [28] have also been reported as carotenoids isolated from shrimp muscle. Although the strongest signals were detected at visible spectra (with the highest absorption detected at 450 nm), few signals at the near and middle ultraviolet spectra were observed.

#### 3.2.1. Antimutagenic Activity of Methanolic Subfractions

The 15 different subfractions were analyzed in order to identify the bioactive fractions with the highest antimutagenic activity. Each fraction was tested for antimutagenicity at a concentration of 4 mg/plate, using 500 ng of AFB_1_ as control mutagen in the Ames test (Table 2).

All tested fractions exerted antimutagenic activity to a certain magnitude; nevertheless, low mutagenic inhibition was detected in M1 sample, and some of the samples were active only on one tester strain such as M3 and M5 fractions. This might be due to the fact that* Salmonella* TA98 and TA100 tester strains are used for two different types of mutagens; TA98 detects various frame shift mutagens whereas TA100 is prone to base-pair substitutions. On the other hand, some fractions exerted high inhibition of AFB_1_ mutagenicity in both bacteria tester strains.

**Figure 3:**
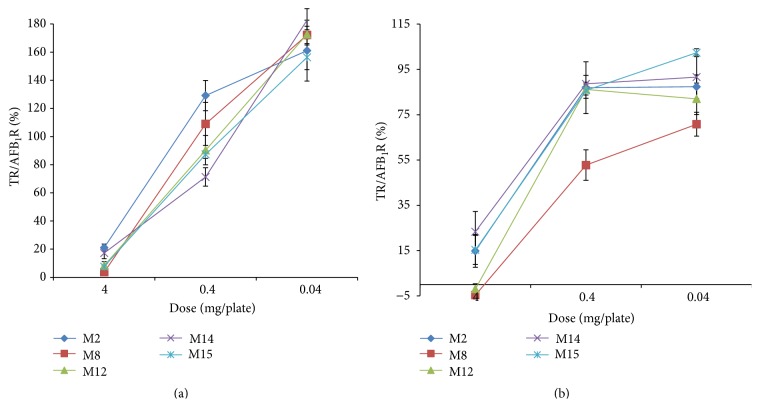
Antimutagenic activity of methanolic subfractions M2, M8, M12, M14, and M15 at different concentrations. Values are the percentage of inhibition of AFB_1_ (500 ng) mutagenicity in* Salmonella* TA98 (a) and TA100 (b) tester strains. Results are representative of three repetitions. Spontaneous revertants were 33 ± 4 and 120 ± 8 and AFB_1_ control (500 ng) induced 493 ± 37 and 724 ± 2 revertants/plate for TA98 and TA100, respectively. TR: number of treatment-induced revertants; AFB_1_R: number of aflatoxin B_1_-induced revertants/plate.

Five subfractions were selected for further analysis including M2, M8, M12, M14, and M15 since all showed high antimutagenic activity in both tester strains (higher than 60% mutagenesis inhibition) [23] without a significant difference among them. Differences in the retention times of these five subfractions indicate that they differ in polarity as well as in chemical structure. Fractions M2, M14, and M15 were all colorless, M8 exhibited an intense orange color, and M12 was pale yellow colored. Lower concentrations of these five subfractions were used to assess their antimutagenic activity (serial dilutions from 4 to 0.04 mg/plate) (Figure 3). All five subfractions exhibited a dose-response type of relationship, and subfraction M8 was selected for further analysis since it showed the highest activity on both tester strains.

### 3.3. Fractionation by Open Column Chromatography

Isolation of the bioactive compounds was continued through M8 fractionation, which was subjected to a low-pressure chromatographic procedure (open column). Four new fractions were obtained, which were coded as M8a, M8b, M8c, and M8d. Polarity of sample decreased as follows: M8d > M8c > M8b > M8a; this last one exhibited a bright orange color; M8b and M8c showed a pale orange tone, whereas M8d had a pale yellow color.

#### 3.3.1. Antimutagenic Activity of Methanolic Subfractions Isolated by Open Column Chromatography

**Figure 4:**
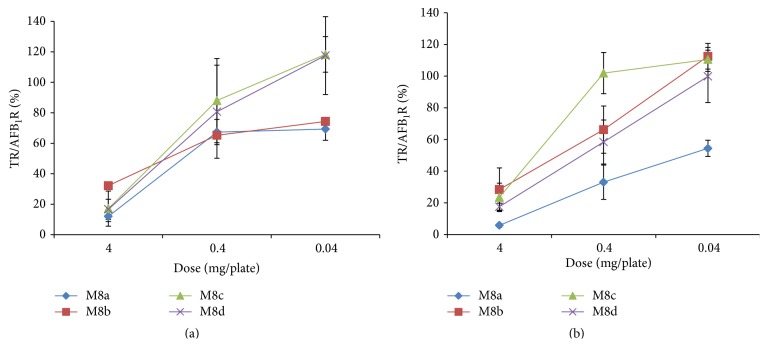
Antimutagenic activity of methanolic subfractions M8a, M8b, M8c, and M8d tested at different concentrations. Values are the percentage of inhibition of AFB_1_ (500 ng) mutagenicity in* Salmonella* TA98 (a) and TA100 (b) tester strains. Results are representative of three repetitions. Spontaneous revertants were 33 ± 4 and 120 ± 8 and AFB_1_ control (500 ng) induced 493 ± 37 and 724 ± 21 revertants/plate for TA98 and TA100, respectively. TR: number of treatment-induced revertants, AFB_1_R: number of aflatoxin B_1_-induced revertants/plate.

All of the M8 subfractions were highly antimutagenic and exerted a dose-response relationship (Figure 4). Since fraction M8a exerted the highest antimutagenic activity in both tester strains (87.9 ± 3.4 and 94.1 ± 1.2% for TA98 and TA100 tester strains, resp.), it was analyzed in its chemical structure.

#### 3.3.2. Chemical/Structural Characterization of M8a Fraction

**Figure 5:**
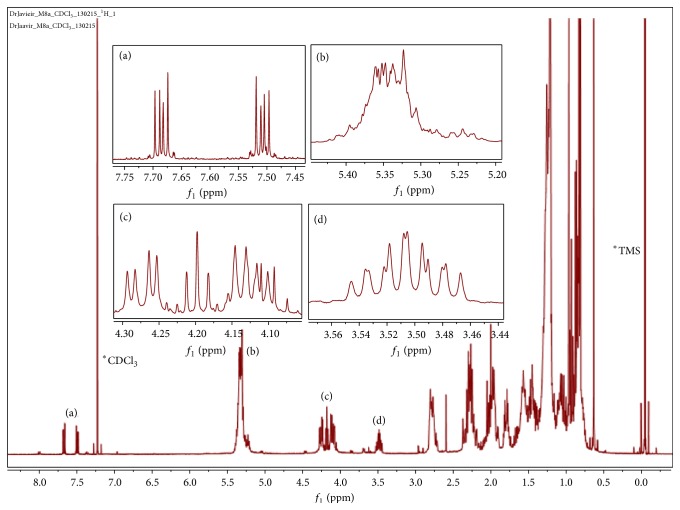
^1^H NMR spectra of M8a subfraction dissolved in CDCl_3_.

According to the ^1^H NMR spectra (400 MHz) (Figure 5), downfield signals at *δ* = 7.5–7.75 ppm are evidences of hydrogen atoms attached to an aromatic ring arranged in the ortho position; however, there is absence of the characteristic signals of carotenoid compounds downfield (*δ* = 6.0–6.7 ppm), which indicates that even though the color of the sample is orange, the compounds are not carotenoid. Signals observed at *δ* = 5.0–5.5 ppm may be attributed to protons involved in double bond, whereas signals at *δ* = 4.2 and 4.5 ppm are associated with protons adjacent to carbons attached to an ester bond (C–O). The signals found at signals at *δ* = 3.5 ppm are associated with protons in alcohol groups. Finally, chemical shifts that appear at high field (*δ* = 0–3.0 ppm) are attributed to methyl, methylene, and methine protons. All of these signals are characteristic of apocarotenoid compounds.

**Figure 6:**
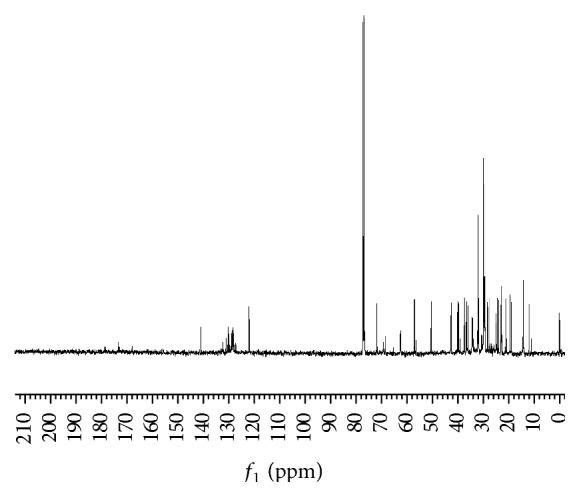
^13^C NMR spectra of M8a subfraction dissolved in CDCl_3_.

This information is corroborated by the ^13^C NMR spectra (400 MHz) (Figure 6), where downfield signals *δ* = 170 ppm indicate the presence of a carbon involved in an ester bond; signals at *δ* = 140 and 120 ppm are evidence of double bonds, whereas a chemical shift in *δ* = 127–133 suggests the presence of aromatic compounds. The chemical shift of *δ* = 77 is attributed to the solvent CDCl_3_ and *δ* = 50–72 ppm is evidence of carbons bound to oxygen atoms, whereas *δ* = 0–50 ppm may be attributed to methyl, methylene, and methine carbons.

The presence of bioactive compounds in shrimp has been previously reported; however, most of them were not extracted from shrimp muscle but from exoskeleton. Biological activities previously reported include antioxidant, which was found in crude extracts obtained from shrimp byproducts such as head [14, 15] and shell [16], and anti-inflammatory activity also on shrimp's shell [16] and antimutagenic activity [17] in muscle crude extracts.

In all of these reports, bioactivity has been attributed to carotenoids, specifically to astaxanthin; nevertheless, all of these studies were carried out on crude extracts only, and their conclusions were based on absorbance observed at visible spectra wavelength (450–475 nm), attributing the bioactivity to carotenoids without any fractionation of the extract in order to isolate and identify the compound responsible for the bioactivity.

Recently, antimutagenic compounds present in fractions obtained after serial thin layer chromatography procedures have been reported [29]. In the present study, the existence of compounds in white shrimp muscle, with the ability to suppress the mutagenic effect of aflatoxin B_1_, has been evidenced; but also the fact that these compounds are not carotenoids has been demonstrated. Results of the present study suggest that products of the breakdown of this type of compounds called apocarotenoids are responsible for the antimutagenic activity found in white shrimp muscle.

Carotenoid breakdown might be either enzymatic- or not enzymatic-type and can produce different kinds of compounds, depending on the reaction conditions. It has been previously reported that biological processes can be affected by these kinds of compounds instead of pure carotenoids, and they are solely responsible for the biological activity reported in carotenoids [30].

Apocarotenoids have previously been reported as bioactive compounds capable of showing bioactive properties; among those, bixin is an apocarotenoid isolated from the shrub* Bixa orellana*, which has been reported as an anticancer compound [31]. Specifically, this apocarotenoid along with norbixin has been reported as an antiproliferative compound effective against melanoma murine cells [32]. Ditaxin and heteranthin, which are apocarotenoids isolated from saffron (*Ditaxis heterantha*), have also been reported as antiproliferative compounds in human malignant cells (HeLa and CaLo) [33]. Another apocarotenoid with anticancer activity is *β*-apo-8′-carotenal, which has been reported as an aflatoxin B_1_ inhibitor in rats [34]. Even though these activities have been reported on apocarotenoid compounds, to our knowledge, there is no previous work reporting apocarotenoids isolated from shrimp as compounds responsible for biological activity.

## 4. Conclusions

The chloroform-soluble fraction from* Litopenaeus vannamei* muscle is a source of different antimutagenic compounds and even though astaxanthin is thought to be responsible for this activity, the present study demonstrated that the compounds that exerted the highest activity have an apocarotenoid chemical structure.

## Figures and Tables

**Table 1:** Antimutagenicity of white shrimp muscle-crude chloroform extract and its methanolic and hexanic fractions tested on *Salmonella typhimurium* tester strains.

Dose (mg/plate)	Crude extract	Methanolic fraction	Hexanic fraction
TA98			
5	94.6 ± 1.1^a^	95.6 ± 0.6^a^	67.8 ± 1.1^b^
0.5	12.6 ± 12.6	31.3 ± 13.8	54.7 ± 14.3
0.05	10.2 ± 12.4	10.2 ± 4.2	−10.2 ± 9.8

TA100			
5	95.3 ± 2.4^a^	95.9 ± 1.9^a^	32.7 ± 8.0^a^
0.5	2.5 ± 14.6	11.5 ± 6.0	0.2 ± 11.1
0.05	−14.5 ± 17.2	−8.6 ± 12.8	−34.9 ± 9.1

Results are presented as the percentage of inhibition of AFB_1_ mutation and are representative of three repetitions.

Values with different letters within a row are significantly different (*P* < 0.05). Spontaneous revertants were 31 ± 3 and 117 ± 6, and AFB_1_ control (500 ng) induced 625 ± 26 and 958 ± 27 revertants/plate for TA98 and TA100, respectively.

**Table 2:** Antimutagenicity of fractions obtained after RP-HPLC fractionation of a methanolic fraction from white shrimp muscle tested on *Salmonella typhimurium* tester strains.

	TA98	TA100
M1	22.8 ± 5.7^a^	27.1 ± 10.2^ab^
M2	66.5 ± 5.1^bc^	66.1 ± 5.6^de^
M3	63.1 ± 10.1^bc^	17.7 ± 8.5^a^
M4	58.6 ± 10.7^abc^	72.5 ± 7.3^de^
M5	70.1 ± 3.6^bc^	31.0 ± 10.7^abc^
M6	66.5 ± 1.2^bc^	42.8 ± 2.1^abcd^
M7	41.1 ± 11.7^ab^	30.9 ± 7.4^abc^
M8	80.0 ± 7.0^c^	63.7 ± 4.6^cde^
M9	40.8 ± 11.7^ab^	53.0 ± 9.3^bcde^
M10	45.0 ± 11.4^abc^	46.59 ± 6.9^abcde^
M11	48.6 ± 9.6^abc^	56.2 ± 11.6^bcde^
M12	68.0 ± 5.5^bc^	79.6 ± 4.3^e^
M13	52.6 ± 11.4^abc^	53.3 ± 2.9^bcde^
M14	74.8 ± 7.4^bc^	59.2 ± 7.2^bcde^
M15	71.9 ± 4.8^bc^	71.0 ± 7.1^de^

Results are presented as the percentage of inhibition of AFB_1_ mutation and are representative of three repetitions.

Values with different letters within a column are significantly different (*P* < 0.05). Spontaneous revertants/plate were 31 ± 3 and 117 ± 6 and AFB_1_ control (500 ng) were 625 ± 26 and 958 ± 27 revertants/plate for TA98 and TA100, respectively.
